# Biogeographical patterns of species richness in stream diatoms from southwestern South America

**DOI:** 10.1002/ece3.11156

**Published:** 2024-03-20

**Authors:** Daniel Zamorano, Fabio A. Labra, Christoph D. Matthaei, Úrsula Romero

**Affiliations:** ^1^ Centro de Investigación e Innovación para el Cambio Climático, Facultad de Ciencias Universidad Santo Tomás Santiago Chile; ^2^ Department of Zoology University of Otago Dunedin New Zealand; ^3^ Programa de Doctorado en Conservación y Gestión de la Biodiversidad, Facultad de Ciencias Universidad Santo Tomás Santiago Chile; ^4^ Laboratorio de Limnología, Facultad de Ciencias Universidad de Chile Santiago Chile

**Keywords:** Clementsian structure, flow velocity, Glasonian structure, latitudinal diversity gradient, number of limiting resources, PATICE, silica

## Abstract

The latitudinal diversity gradient (LDG) hypothesis has been validated for many taxon groups, but so far, stream diatoms have not conformed to this pattern. Research on diatoms that includes data from South America is lacking, and our study aims to address this knowledge gap. Previous studies have successfully explained stream diatom species richness by considering niche dimensionality of physicochemical variables. Moreover, in southwestern South America, the observed biogeographical pattern differs from LDG and has been shown to be determined by historical factors. We used a dataset comprising 373 records of stream diatom communities located between 35° S and 52° S latitude, southwestern South America. The dataset included physicochemical river water variables, climate data, and ice sheet cover from the Last Glacial Maximum. We explored geographical patterns of diatom species richness and evaluated 12 different causal mechanisms, including climate‐related theories, physicochemical and climatical exploratory analyses, historical factors, and niche dimensionality. A metacommunity analysis was conducted to evaluate the possible nested structure due to historical factors. We observed an increase in diatom species richness from south to north. Models containing both physicochemical and climatic predictors explained the highest proportion of variation in the data. Silica, which was correlated with latitude, and flow velocity, which did not show any spatial pattern, were the most important predictors. Historical factors and nested structure did not play any role. Contrary to what has been reported in the literature, we found no support for climate‐related explanations of species richness. Instead, theories related to niche dimensionality and local factors provided better explanations, consistent with previous related research. We suggest that the increase in diatom richness in the north of our study region is due to a higher nutrient supply in these rivers, rather than a due to larger species pool in the area.

## Graphical abstract

We assessed biogeographical patterns in riverine diatom species richness using a dataset from southwestern South America. Diatom species richness increases towards the north and is explained by local physicochemical variables and the Niche dimensionality hypothesis, while classical climate‐related theories and historical variables exhibit low explanatory power.

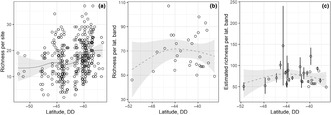

## 1 INTRODUCTION

The latitudinal diversity gradient (LDG) hypothesis, which predicts an increase in species richness from the poles to the Equator, has been widely tested (Brown, 2014; Evans et al., 2005; Hillebrand, 2004; Hubbell, 2005; Passy, 2010; Pianka, 1966; Presley et al., 2010; Von Humboldt, 1807). A LDG has been recorded for a broad range of organisms, including terrestrial vertebrates (Currie, 1991), terrestrial plants (O'Brien, 1993), marine and freshwater fish (Guégan et al., 1998; Stevens, 1996), marine bivalves (Jablonski et al., 2013), and many others (Hillebrand, 2004; Willig et al., 2003). This gradient has even been observed in fossil records (Crame, 2001) and in human cultures and languages (Collard & Foley, 2002; Gavin et al., 2013; Pagel & Mace, 2004). Nevertheless, certain organism groups do not conform to this pattern, such as aquatic angiosperms (Crow, 1993), hymenopterans (Price et al., 1998; Skillen et al., 2000), penguins and seals (Procheş, 2001), seaweeds (Santelices & Marquet, 1998), marine peracarids, (Doti et al., 2014; Rivadeneira et al., 2011), marine polychaetes (Hernández et al., 2005; Moreno et al., 2006), and some others (Kiel & Nielsen, 2010; Kindlmann et al., 2007; Willig et al., 2003). These exceptions to the rule suggest that further research on the LDG and proposed theories is needed.

One of the groups that has shown no LDG are stream diatoms (Hillebrand, 2004; Soininen et al., 2016). At a global scale, a metanalysis by Hillebrand (2004) found no significant relationship between latitude and freshwater algal species richness. However, at a slightly smaller scale, Soininen et al. (2016) found an increase of diatom richness from mid‐southern‐hemisphere latitudes (40° S) to northern latitudes (60° N). At a continental scale, Passy (2010) found that diatom richness decreased between 30° and 40° N in the USA, whereas Passy et al. (2018) observed no clear latitudinal pattern of diatom richness across the entire USA (25°–62° N) but found a longitudinal pattern of richness instead. Moreover, in the same study data from Finland (60°–70° N) showed that diatom richness peaked at intermediate latitudes (66° N). Thus, the current literature does not show a consensus regarding the existence of LDGs, or any other consistent latitudinal biogeographical pattern, for stream diatoms. Hence, additional research is needed to increase our understanding of biogeographical patterns in stream diatoms, and the drivers that explain their species richness. One of the aims of the present paper is to test the applicability of the LDG hypothesis to stream diatoms by studying, for the first time, a database from southwestern South America.

The debate on the causal processes behind the LDG remains open (Brown, 2014; Currie et al., 2004; Hawkins et al., 2003; Hwang et al., 2009; Kindlmann et al., 2007; Qian & Ricklefs, 2016; Willig et al., 2003). Willig et al. (2003) synthesized the hypotheses developed to that date to explain the LDG and listed 30 of them, an impressive number. While some are circular and others insufficiently supported, most of the existing hypotheses are backed up with enough evidence based on different taxon groups, including terrestrial plants, mammals, amphibians, or arthropods (Currie, 1991; Currie et al., 2004; Jiménez‐Alfaro et al., 2016; Rodríguez et al., 2005; Ulrich et al., 2016). A recent study by Passy et al. (2018) aimed to test these hypotheses in stream diatoms, but reported results that contrasted with those expected based on the relevant literature. While variation in diatom richness in Passy et al.'s study was explained by local (physicochemical river variables) and climatic variables (i.e. air temperature), as documented in many other taxa (Coops et al., 2019; Jiménez‐Alfaro et al., 2016; Moura, 2017), the observed biogeographical patterns did not fit the LDG or predictions derived from species‐energy theory (Wright, 1983), metabolic theory (Allen et al., 2002; Brown et al., 2004) or the climatic tolerance hypothesis (Currie et al., 2004). In a different approach, Passy (2008) proposed another hypothesis for benthic algal communities, which has been able to successfully explain algal species richness through the number of limiting resources (NLR) (Larson et al., 2016; Passy, 2008; Passy & Larson, 2019). According to Passy (2008), high resource supply favors species that extend above the substrate and develop algal mats where other tolerant species can persist, thus increasing overall species richness. This hypothesis allows explaining diatom richness patterns using a model based on the dynamics and structure of freshwater ecosystems, without resorting to classical, climate‐based biogeographical hypotheses.

In our study area, the southwestern area of South America (Chile), a biogeographical pattern that is in contrast to LDGs has been well‐documented for many taxon groups, with an observed unimodal species richness gradient that peaks around 38° S for small mammals (Cofré et al., 2007), testate amoebas (Fernández et al., 2016), trees (Segovia et al., 2013; Villagrán & Hinojosa, 1997), and freshwater fish (Vila et al., 1999). This richness peak has been spatially constrained by historical and climatic factors (Segovia et al., 2013). To the north of this peak, the Atacama Desert (17°–28° S) has very low rainfall and high temperatures that were accentuated during the Early Pleistocene (Clarke, 2006). To the south, a series of glacial advances and retreats modified the landscape, and during the Last Glacial Maximum in the Middle Pliocene an ice sheet almost completely covered South America between 38° and 56° S (Villagrán & Hinojosa, 1997, 2005). These drastic climatical changes have driven extinctions and several migrations around 38° S (Central Chile) (Armesto et al., 2010), resulting in a biodiversity hotspot with high diversity and endemism (Myers et al., 2000). Particularly in the latitudinal range we assess (35–52° S), it has been established that the movements of ice sheets resulted in interglacial refuges for multiple species, being evidenced through populations with high levels of genetic diversity (Marín et al., 2013; Premoli et al., 2010; Segovia et al., 2012; Sepúlveda‐Espinoza et al., 2022). Consequently, sites covered by ice sheets during the Last Glacial Maximum should exhibit lower species richness, comprised by a subset of the species present at refuge sites (Nestedness), due to recolonization processes after the Last Glacial Maximum (Srinivasan et al., 2014; Valdovinos et al., 2010). Thus, when considering the evaluation of LDG hypotheses, the southwestern South American biogeographical context provides a unique opportunity to assess the importance of historical factors to account for geographical variation in stream diatom richness patterns.

The overall aim of our study was to evaluate a set of competing hypotheses to account for biogeographical patterns of stream diatom species richness, thus testing the support for the classical LDG, while also considering theoretical frameworks for stream ecosystems and local hypotheses specific to the Chilean biodiversity hotspot, using a novel taxonomic group for the region. We addressed this aim by testing and comparing the explanatory power of 12 different hypotheses. These can be grouped into five exploratory analyses and seven formal hypotheses, composed for spatial, physicochemical, climatical, and historical variables (Table 1). We started by applying a set of phenomenological hypotheses or models to evaluate the relationship between diatom richness and predictor variables, which could either show the existence of geographical patterns or associations that were consistent with the presence of ecological limits. Thus, the first two models explored the presence of spatial patterns either through latitude alone, or through latitude, longitude, and elevation. The next hypothesis tested whether climatical temperature variables showed significant relationships with diatom species richness, as studies on freshwater diatoms elsewhere have shown significant but inconsistent relationships (Jamoneau et al., 2022; Passy et al., 2018; Soininen et al., 2016). The next hypothesis examined the potential effects on species richness of ecological limits in the water column, by determining relationships with physicochemical predictor variables. In this context, there is a substantial literature documenting significant relationships between benthic algal richness and/or productivity with several physicochemical and nutrient supply variables (Chetelat et al., 1999; Hill et al., 2003; Passy, 2010; Passy et al., 2018; Soininen et al., 2016; Tonkin et al., 2014; Yuan et al., 2023). While most of these studies focused on linear relationships, unimodal patterns based on subsidy‐stress theory have also been observed (Biggs et al., 1998; Hart et al., 2013; Wagenhoff et al., 2011), making it necessary to evaluate these predictors case by case. Finally, we applied a model that simultaneously included historical, climatical, and physicochemical parameters, allowing us to compare them in the same statistical context.

**TABLE 1:** List of hypotheses and models used to assess competing biogeographical theories.

Hypothesis categories	Model tested	Mechanism	Complete dataset predictors (373 data points)	Chemical dataset predictors (182 data points)	Expected relationship with diatom richness
Phenomenological and ecological limits hypotheses	Latitudinal effect	Phenomenological correlation with latitude	~ Lat	~ Lat	Positive or unimodal
	Spatial effect	Geographical or spatial patterns result in observed variation	~ Lat + Lon + Elev	~ Lat + Lon + Elev	Linear or unimodal
	Climatic effects	Overall variation in temperature regimes limits the distribution of species richness	~ T_min + T_max + T_sd + T_mean	~ T_min + T_max + T_sd + T_mean	Linear or unimodal
	Water physicochemical limits	Water physicochemical variables define a set of multidimensional constraints on species distribution and as a result on species richness	N/A	~ T_insitu + pH + CE + Osat + Ca + TP + SiO_2_ + Vel	Linear or unimodal
	Full model	Combined model considering all the previous causal variables	~ T_min + T_max + T_sd + T_mean + Glac + Fglac	~ T_insitu + pH + CE + Osat + Ca + TP + SiO_2_ + Vel + T_min + T_max + T_sd + T_mean + Glac + Fglac	Linear or unimodal
Formal hypothesis	Species‐energy theory via temperature	Energy availability determines higher species richness but also population sizes (Wright, 1983)	~ T_mean	~ T_mean	Positive
	Species‐energy theory via cell density	Energy availability determines higher species richness but also population sizes (Wright, 1983)	~ ln(Cel_den + 1)	~ ln(Cel_den + 1)	Positive
	Energy variability hypothesis	Energy variability explain species richness patterns because periods of low temperatures support fewer individuals that are prone to higher extinction rates (Carrara & Vázquez, 2010)	~ T_sd	~ T_sd	Negative
	Climatical tolerance hypothesis	The tropics should have higher species richness because their minimal and maximal climate conditions are more benign (Currie et al., 2004)	~ T_min + T_max	~ T_min + T_max	Positive to T_min, negative to T_max
	Metabolic theory	Higher energy promotes higher metabolic and mutation rates, leading to shorter speciation time and higher species richness (Brown et al., 2004)	~ 1/k*T_mean	~ 1/k*T_mean	Positive
	Historical effect	The movements of ice sheets during the Last Glacial Maximum resulted in interglacial refuges for multiple species in northern areas of southern South America (Segovia et al., 2012)	~ Glac + Fglac	~ Glac + Fglac	Negative to Glac, higher on Fglac = 1
	Niche dimensionality	Diatom biodiversity in streams declines as more nutrients become limiting because of lack of biofilm production (Passy, 2008)	N/A	~ Number of limiting resources (NLR)	Negative

*Note*: We employed two different datasets: Complete dataset (with all sites included) and Chemical dataset (only sites with full water physicochemical data). For the Complete dataset, equations containing physicochemical variables were not evaluated. The column “Expected relationship with diatom richness” indicates the expected relationship between diatom species richness and the predictors. For more details, see Section 2.

Abbreviation: N/A, not applied.

The second set of hypotheses examined specific biogeographical hypotheses proposed in the literature (Table 1). Species‐energy theory suggests energy is proportional to the population sizes and hence to the species richness (Carrara & Vázquez, 2010; Wright, 1983). In our study, we evaluated this theory using two equations: one examined the effect of temperature and the second examined possible relationships with total cell density. The Energy variability hypothesis is a modification of the Species‐energy theory that includes seasonal energy variation. This hypothesis suggests that low‐energy periods constrain population abundance and increase probability of extinction, thus energy variability should lead to lower species richness (Carrara & Vázquez, 2010). On the other hand, the Climatic tolerance hypothesis suggests that, at sites with extreme climatic conditions (too cold, too hot), species will require more physiological adaptation to tolerate these conditions and persist, thus decreasing species richness (Currie et al., 2004). The next hypothesis examined is based on the metabolic theory of ecology (MTE), which suggests that higher energy promotes higher metabolic and mutation rates, leading to shorter speciation time and increasing species richness (Brown et al., 2004). The Historical effect hypothesis evaluated the role of ice sheets during the Last Glacial Maximum, and predicted that sites never covered by ice sheets should exhibit higher species richness, due to acting as interglacial refuges (Segovia et al., 2013). Finally, the Niche dimensionality hypothesis predicts that habitats with higher niche dimensionality (lower number of limiting resources – NLR) should sustain higher species richness due to the production of algal mats developed by eutrophic species (Passy, 2008).

We further expected that, if historical factors are driving the stream diatom metacommunity, it should exhibit a Nested structure (Patterson & Atmar, 1986), where the species pool of southern and high elevation sites would be nested within the species pool of northern and low elevation sites. Besides a Nested structure, evaluating stream diatom metacommunity structure also allows us to test other metacommunity structures such as Gleasonian or Clementsian. A Gleasonian structure describes a metacommunity composed by species following their environmental requirements (Gleason, 1926), for example, their tolerances to climate or local physicochemical conditions. A Clementsian structure describes a clustered metacommunity whose species share a common evolutionary history or inter‐dependent ecological relationships (Clements, 1916). Such clustered metacommunities could be the result of hydromorphological boundaries or spatially clustered or autocorrelated environmental variables.

Our study complements the existing biogeographical literature by providing new data from 17 distinct river catchments located adjacent to each other along a nearly straight north‐to‐south gradient spanning 17° degrees in southwestern South America. These catchments are situated within a well‐documented and similar biogeographical context, thus offering an ideal situation to generate new insights into stream diatom biogeography, a novel taxonomic group for that region. We complemented the models proposed in Table 1 with exploring LDG using samples‐size‐corrected richness and latitudinal‐band richness, metacommunity structure evaluation (Nested, Gleasonian or Clementsian), and further statistical analyses, aiming for a deeper understanding of our dataset.

## 2 MATERIALS AND METHODS

### 2.1 Database

**FIGURE 1:**
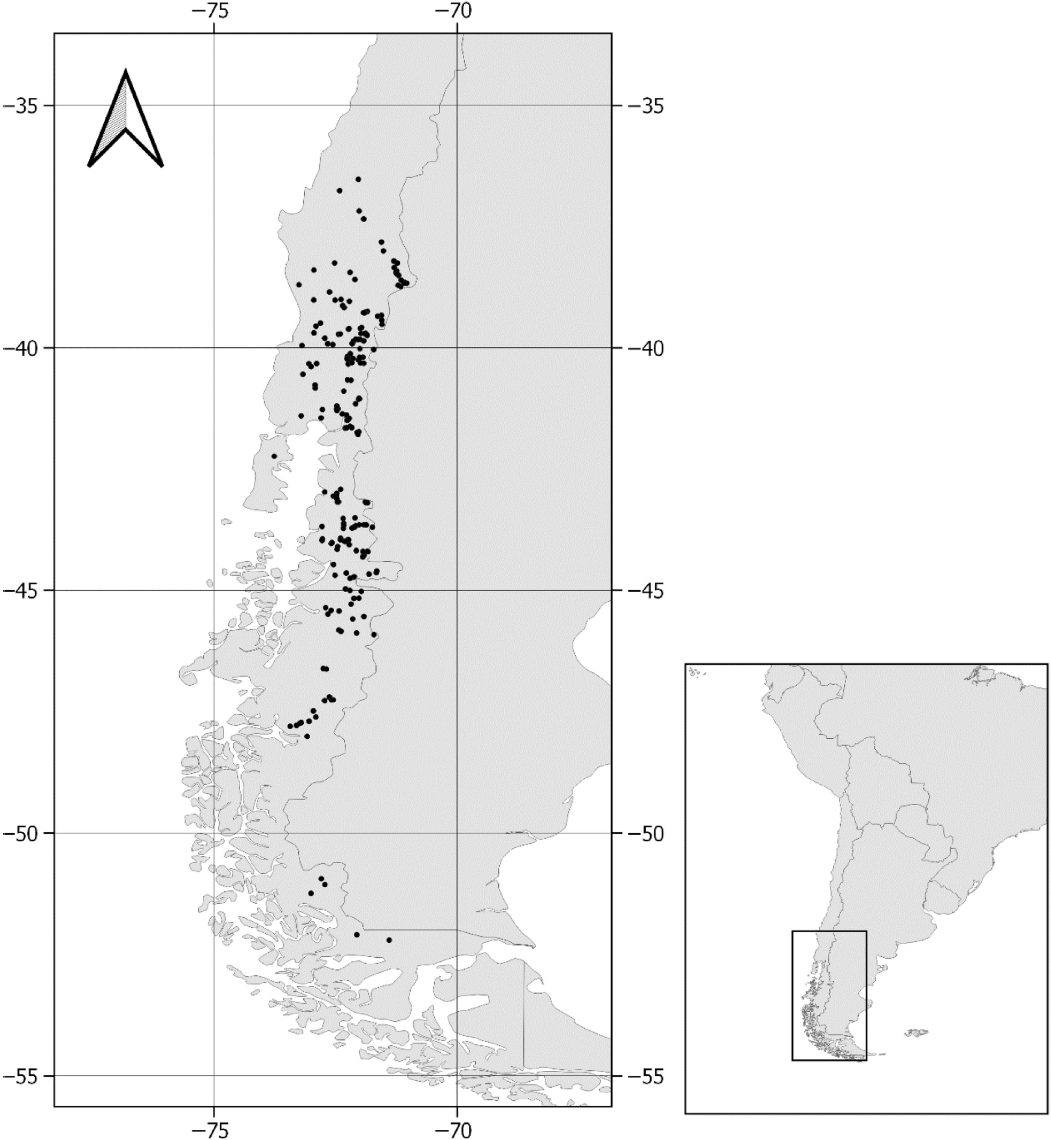
Spatial distribution of the 373 study sites. Filled circles show the geographical distribution of the sampling locations in southern Chile. The inset map shows the location of the study area in South America.

Our dataset comprised 373 records of benthic diatom communities distributed across 246 southern Chilean rivers. These studies were carried out between 2010 and 2014 in 20 river catchments located between 35° S and 52° S latitude and 70° W and 75° W longitude (Figure 1). At every study site, a periphyton sample was obtained and several physicochemical parameters were measured (see details below). These data were obtained during several Chilean government monitoring schemes, and were verified and systematized for Servicio Nacional de Pesca y Acuicultura (2014) (National Service of Fisheries and Aquaculture).

### 2.2 Field and laboratory procedures

The periphyton community at each study site was sampled using a standard methodological procedure, mandated by the government project terms of reference (Díaz et al., 2012). At each site, a reach of around 20 m length was chosen, and five randomly selected surface stones (one every 4 m) were sampled. At sites with periphyton mats thicker than 1 mm, one biofilm sample of 1 mL volume was taken from each upper stone surface utilizing a dispensable blunt syringe. At sites with thinner mats, an upper surface area of 4 cm^2^ on the selected stones was scrubbed with a toothbrush. In either case, a single composite sample was created per site by pooling the individual samples. All samples were preserved in the field with 4% formaldehyde solution and stored in fixed 15‐mL holders.

In the laboratory, diatom samples were prepared and processed following the methodology described by Balech and Ferrando (1964) and Battarbee (1986). Taxonomic identification and frustule counts were carried out under an Axiostar II Plus Zeis microscope (1000×). Counts comprised at least 300 valves per sample (Battarbee, 1986). Specimen identification was done to the species level, relying on extensive available literature (Archibald, 1983; Hartley, 1996; Krammer & Lange‐Bertalot, 1986, 1991; Krasske, 1939; Lange‐Bertalot, 1993, 1999, 2000, 2001; Lange‐Bertalot & Krammer, 1989; Lange‐Bertalot & Metzeltin, 1996; Metzeltin et al., 2005; Rivera, 1983; Round et al., 1996; Round & Bukhtiyarova, 1996; Rumrich et al., 2000; Simonsen, 1987).

### 2.3 Predictor variables

At each study site, several physicochemical parameters were determined upstream of each periphyton sampling location. Water temperature in situ (T_insitu), pH, electrical conductivity (EC), and oxygen saturation (Osat) were estimated in situ, using a HANNA Model HI 9828 multiparameter portable meter. Further, water depth above each sampled stone (depth) was determined using a ruler, and near‐bed flow velocity close to each stone was measured (Vel) using a Global Water FP 101 digital flow meter. These depth and flow velocity values were averaged for each study reach. In addition, three water samples of 1 L each were taken and analyzed in the laboratory to determine calcium (Ca), total phosphorus (TP), total nitrogen (TN), and silicate concentrations (SiO_2_) following standard protocols (APHA, 2005). TN concentrations were mostly below the detection limit; therefore, this nutrient was not included as a predictor variable. For statistical analysis, any other physicochemical values lower than the detection limit were transformed to a value between 0 and the detection limit for each variable (Helsel & Cohn, 1988). Because physicochemical data from these laboratory analyses were not available for all 373 study sites (henceforth called ‘Complete’ dataset), the statistical analyses that included these variables relied only on 182 sites (henceforth ‘Chemical’ dataset).

Niche dimensionality was estimated via the number of limiting resources (NLR) in the environment. This number is related to the opportunities for competitive trade‐offs increasing species richness and has been suggested to represent a proxy of niche dimensionality (Harpole & Tilman, 2007; Passy, 2008). NLR was estimated using Ca, TP, SiO_2_ and EC, the latter being a proxy of basic cations (Zinabu et al., 2002). To approximate NLR, we estimated quantiles 0.33 and 0.66 for each variable, assigning a value of 0 to all variables with values above quantile 0.66, 1 to values between quantile 0.33 and 0.66, and 2 to values below quantile 0.33. Values from all variables were then added, resulting in a rank scale between 8 (highest NLR, strong resource limitation) and 0 (lowest NLR, no resource limitation).

Climatical data for each sampled point were acquired using with Worldclim (Fick & Hijmans, 2017), using raster maps with a resolution of around 1 km^2^. Annual mean temperature (T_mean) was obtained from Bio1, maximum temperature of the warmest month (T_max) from Bio5, minimum temperature of the coldest month (T_min) from Bio6, and temperature seasonality (T_sd) from Bio4.

To evaluate the historical role of glaciation, we used a covering map of the ice sheet during the Last Glacial Maximum from PATICE, a GIS database of Patagonian glacial geomorphology (Davies et al., 2020). PATICE includes a geographical reconstruction of the Patagonian ice sheet at 35, 30, 25, 20, 15, 13, 10, 5, and 0.2 ka. Using PATICE, we extracted two variables: the presence or absence of ice sheet during the last maximum glacial per site (Fglac), and the proportion of time under ice sheet during the last 35 ka per site (Glac). The proportion of time under ice sheet was estimated by dividing the age of the last record of ice sheet cover by 35 and subtracting 1. Values for this variable ranged from 0 for sites never under an ice sheet to a maximum of 0.714 for sites whose last ice sheet was present 10 ka ago (1–10/35 = 0.714).

### 2.4 Exploring the LDG gradient

To explore LDG in detail, we evaluated the relationship between diatom species richness and latitude using richness per site and richness per latitudinal band, allowing deeper identification of latitudinal patterns. Latitudinal band widths were fitted to include 12 sites per band, but they were manually modified to also achieve a representative latitudinal extension. The resulting latitudinal bands had a median extension of 0.15° S (range 0.04°–0.37° S) and included between 9 and 18 sites. The only exception was the southern latitudinal band, which included only five sites far distant from the rest that had to be grouped by themselves. This band was also the longest, with 1.3° S of extension. Species richness per latitudinal band was the total of taxa recorded in all sites within the band. The latitude predictor value for each latitudinal band was calculated by averaging the latitude of all sites within the band.

A possible bias due to differences in the number of sites between latitudinal bands was addressed using a sample‐size‐based richness estimator (Chao et al., 2014), implemented through the *iNEXT::iNEXT* algorithm (Hsieh et al., 2016). This algorithm estimates rarefaction curves as a function of sample size and allows for the rarefaction or extrapolation of species richness. Here, we applied this algorithm to extrapolate species richness for each latitudinal band using a common sample size. This software was not applied to correct sites species richness because sampling protocols consistently homogenized their sample effort, and latitudinal bands show variation in the number of sampling sites. Diatom species richness for all sites, and total species richness and estimated species richness for latitudinal bands, were used to evaluate latitudinal patterns using General Additive Models (GAM) (Wood, 2011, 2021). All statistical analyses were conducted in R (R Core Team, 2019).

### 2.5 Models for exploratory analyses and formal hypotheses

We evaluated the relationships between species richness and a suite of different predictors, using all the equations shown in Table 1. We fitted General Additive Models (GAM) using the *mgcv::gam* function in R (Wood, 2021) to evaluate these equations, after applying smooth‐spline transformations on predictors using the *mgcv::s* function (Wood, 2003). These transformations allowed us to explore a broader range of responses than linear models, by evaluating biogeographical hypotheses and conducting exploratory analyses for environmental variables within the same analysis. The smoothing parameter estimation method was REML (Residual maximum likelihood).

The high number of predictors and the limited number of replicates for many equations led us to apply a Null‐space penalization method in the *gam* function. This penalization dropped from the model those predictors whose smoothing parameters did not fit successfully, thereby decreasing their participation in the final model (Marra & Wood, 2011). Hereafter, we comment only on results for predictors with significant contributions to the final model (*p* < .05). Further, a pair‐wise correlation matrix for all predictors and response variables was calculated, aiming to identify potential collinearity problems and obtain a general overview of all statistical correlations. None of the predictors showed very strong correlations (*r* ^2^ > .90), thus no centering of predictor variables was required (Quinn & Keough, 2002). This correlation matrix is displayed in Section 3.

Predictors for all models are presented in Table 1. Latitudinal and Spatial effect models considered as predictors only the latitude coordinates (Lat), or latitude, longitude (Lon) and elevation (Elev). The Climatical effect model included all climatical variables as predictors, and the Chemical effect model included all physicochemical variables as predictors. The latter model was applied only to the Chemical dataset, with all physicochemical variables as predictors. The Total effect model included all variables (physicochemical + climatical + historical) as predictors for the Chemical dataset, but only climatical and historical predictors for the Complete dataset.

In all climate‐based hypotheses, energy was evaluated using temperature variables. Because the species‐energy theory posits that energy availability determines higher species richness but also population sizes (Wright, 1983), we evaluated this theory with two models: one with mean temperature (T_mean), and one with cell density (Cel_den). In the Energy variability hypothesis model (Carrara & Vázquez, 2010), energy variability was expressed using T_sd. For the Climatical tolerance hypothesis, we used the minimal temperature of the coldest month (T_min) and the maximal temperature of the warmest month (T_max) to characterize the most extreme conditions. The Metabolic theory of ecology (MTE) (Allen et al., 2002; Brown et al., 2004) is evaluated by transforming the temperature variable into an energy variable. In this regression, richness was used with natural‐log transformation to linearize its response, which theoretically should be exponential. In this case, the energy predictor was not smooth‐transformed because this theory it is very explicit about the linear relationship.

The Historical effect model evaluated the role of the Last Glacial Maximum in the studied rivers by using the presence or absence of an ice sheet in the Last Glacial Maximum for each site (Fglac), and the proportion of time under an ice sheet during the last 35 ka (Glac). Finally, the Niche dimensionality model (Passy, 2008) included only the Number of Limiting Resource (NLR) as predictor and was estimated solely for the Chemical dataset. The variables Glac and NLR were not smooth‐transformed in any equation because their low numerical variability caused problems for the *mgcv::s* function.

All models were assessed and compared by considering the proportion of deviance explained, as well as their performance according to the Akaike Information Criterion (AIC). Models with the lowest AIC and with an AIC <2 units higher than the lowest one were considered to have the best performance (Burnham & Anderson, 1998).

### 2.6 Metacommunity analyses

We included a metacommunity analysis to evaluate its fit to idealized structures, such as Gleasonian, Clementsian, or Nested structures, among others. To statistically test how close our metacommunity was related to some idealized structure, we applied the *metacom::Metacommunity* function (Dallas, 2014) to obtain the coherence, boundary clumping, and turnover parameters. These parameters were applied to the diagram presented in Presley et al. (2010), which allowed us to identify the closest idealized structure to our data. To carry out the metacommunity analysis, we transformed our community matrix to presence–absence data and omitted singleton taxa, following the suggestions in the function *metacom::Metacommunity*. We carried out 1000 simulations and applied the “swap” method, which is the most conservative option offered in this function.

To obtain the above mentioned structure parameters, the *metacom::Metacommunity* function reordered the community matrix via reciprocal averaging, grouping along the matrix diagonal sites with similar communities and species with similar ranges (Gauch, 1982). To allow a more thorough interpretation, the order of each site in the reordered matrix, which represents a structuring gradient (Dallas, 2014), was related to latitude and species richness using Pearson correlations.

## 3 RESULTS

### 3.1 Spatial patterns

**FIGURE 2:**
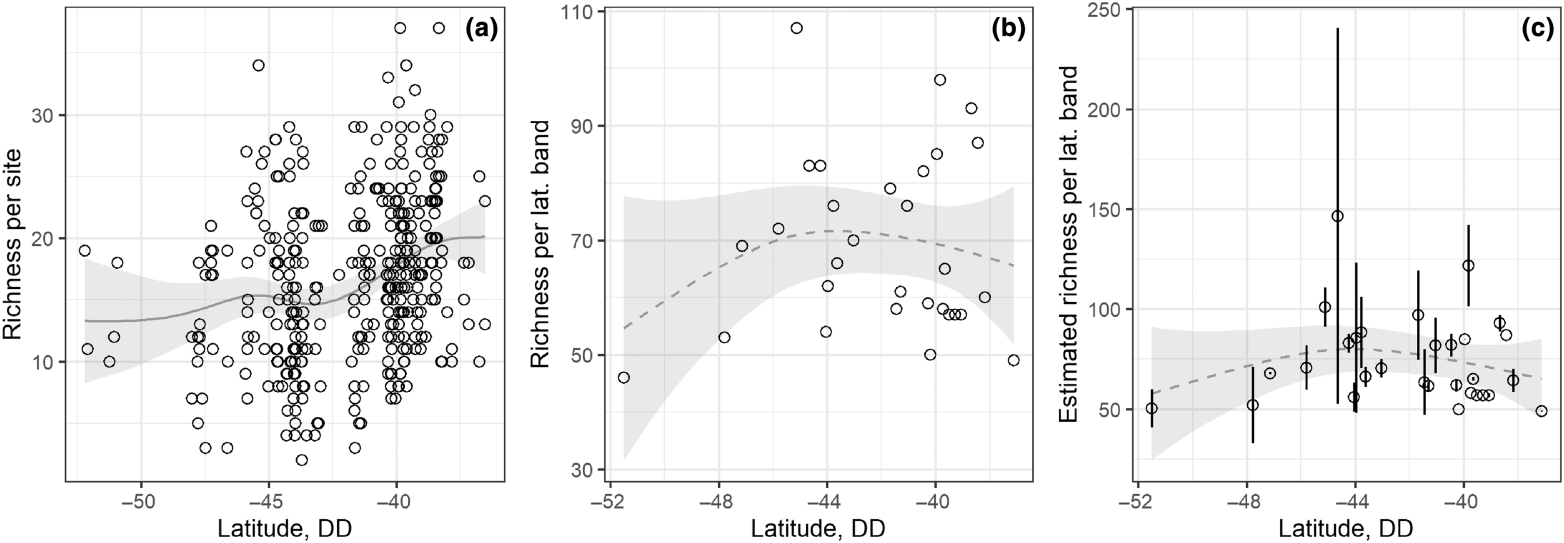
Species richness and estimated species richness in relation to latitude (DD = decimal degree) for the Complete dataset (373 points). (a) Species richness per site. (b) Species richness per latitudinal band. (c) Estimated species richness and confidence interval for each latitudinal band. The smoothed line represents the GAM prediction for the “richness ~ latitude” model, and the gray area is its confidence interval. Solid line = significant model. Dashed line = non‐significant model.

We identified an increase of diatom species richness per site from south to north (Figure 2a), with a richness peak at around 40° S. The GAM significantly fitted a spline‐smooth for species richness per site in function to latitude (Estimated degrees of freedom‐edf = 3.6, *F* = 4.1, *p* = 2.2e‐08). For latitudinal bands, species richness and estimated species richness showed an slight unimodal distribution, with a peak around 44° S (Figure 2b,c). However, GAM did not find significative model between species richness and latitude (edf = 1.95, *F* = 0.77, *p* = .42) or estimated species richness and latitude (edf = 1.89, *F* = 1.05, *p* = .35).

Regarding spatial models, for the Complete and Chemical datasets Latitude showed significant relationships in both Latitudinal and Spatial effect models (Table 2) and including an additional spatial parameter in Spatial effect model did not improve model results. For example, for the Complete dataset, Elevation and Latitude showed significant results but this model indicated a similar deviance explained and AIC as Latitudinal effect model. For the Chemical dataset, Longitude and Latitude showed significant results in Spatial effect model. This model explained a higher deviance than Latitudinal effect model, but still with a higher AIC. Consequently, Latitude was the most relevant spatial predictor variable for diatom species richness in our dataset.

**TABLE 2:** GAM results for the Complete dataset (All; *n* = 373) and Chemical dataset (only sites with full water physicochemical data; Chem, *n* = 182).

Dataset	Model group	Equation name	Significant predictors	Deviance explained %	AIC
All	Exploratory model	Latitudinal effect	Lat	9.86	2441.28
		Spatial effect	Elevation + Lat	9.56	2441.02
		Climatical effect	T_max + T_sd + T_mean	**14.12**	**2434.24**
		Total effect	T_max + T_sd + T_mean	**14.51**	**2435.09**
	Formal hypothesis	Species‐energy theory via temperature	N.S.	0.83	2471.17
		Species‐energy theory via cell density	Cell_den	**9.87**	**2436.06**
		Energy variability hypothesis	T_sd	5.07	2458.66
		Climatical tolerance hypothesis	T_min + T_max	11.91	2439.79
		Metabolic theory	1/k*T_mean	1.56	485.07^a^
		Historical effect	N.S.	5.24	2456.79
Chem	Exploratory model	Latitudinal effect	Lat	8.97	1154.39
		Spatial effect	Lat + Lon	12.71	1147.39
		Climatical effect	T_min + T_max + T_mean	15.51	1144.78
		Chemical effect	T_insitu + pH + Osat + SiO_2_ + Vel	29.92	1117.83
		Total effect	T_insitu + pH + T_max + T_mean + Osat + SiO_2_ + Vel	**40.61**	**1100.17**
	Formal hypothesis	Species‐energy theory via temperature	N.S.	0.00	1167.21
		Species‐energy theory via cell density	Cell_den	13.23	1143.32
		Energy variability hypothesis	T_sd	6.22	1157.36
		Climatical tolerance hypothesis	T_min + T_max	14.85	1145.67
		Metabolic theory	N.S.	0.05	190.69^a^
		Historical effect	N.S.	3.46	1164.80
		Niche dimensionality	NLR	7.86	1154.31

*Note*: Each row provides a summary of the model/hypotheses tested (significant variables included, deviance explained, AIC). For each dataset, the best model or models according to the AIC are indicated in bold. The best models had an AIC of <2 units above the lowest AIC.

Abbreviation: N.S., no significant predictor variables.

^a^ Non‐comparable AIC because the response variable (species richness) was ln‐transformed due to the specific aim of the equation in question.

### 3.2 Correlation matrix

**FIGURE 3:**
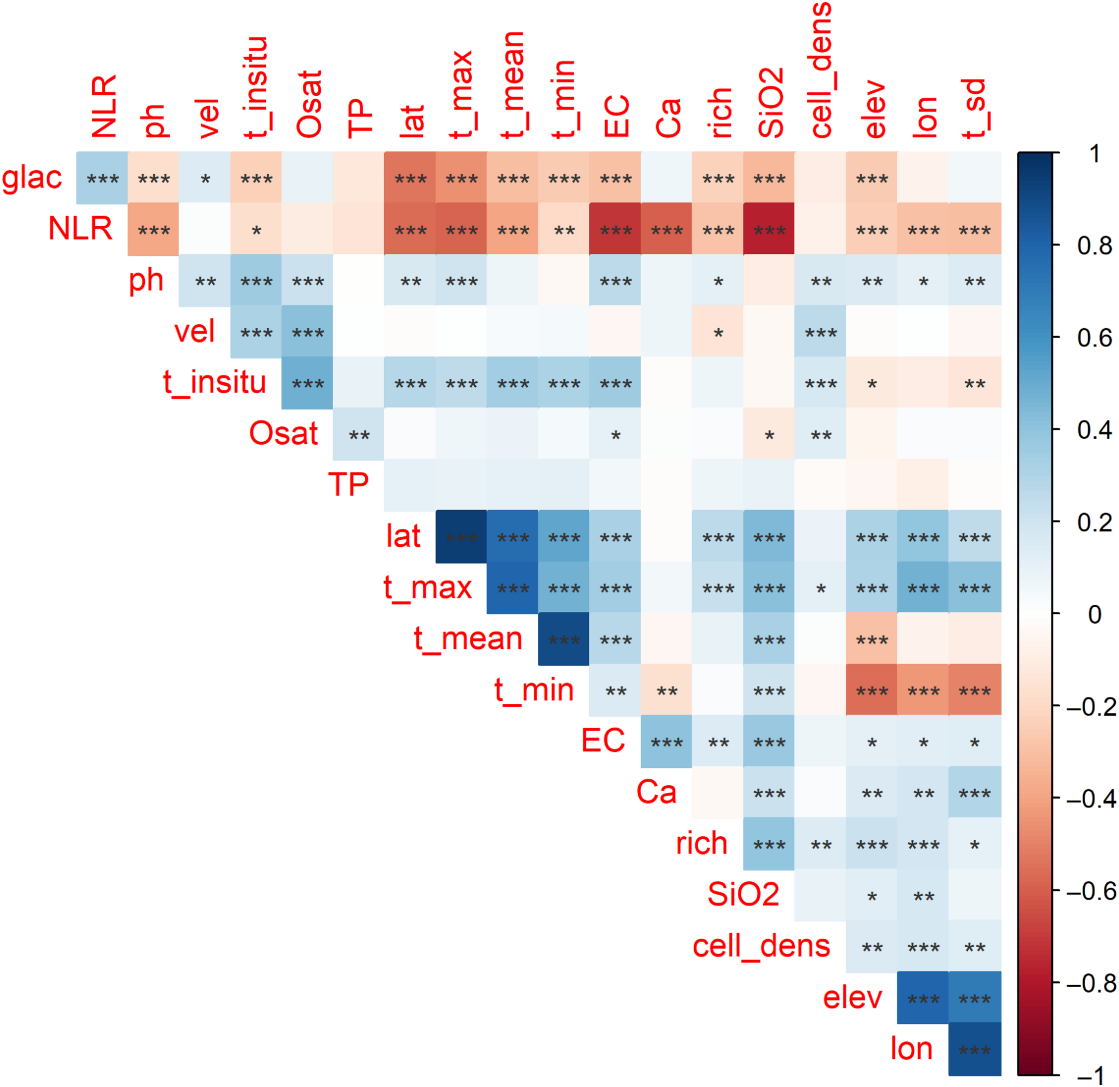
Correlation matrix between predictor variables based on correlation coefficient r (Gradient color) and correlation significance following Pearson tests (***p < .001; **p < .01; *p < .05). Species richness = Rich. For spatial variables: Lat = latitude, Lon = longitude. For climatical variables: T_mean = Annual mean temperature, T_max = Maximum temperature during the warmest month, T_min = minimum temperature of coldest month, T_sd = temperature seasonality. For physicochemical variables: T_insitu = In situ temperature, EC = Electrical conductivity, Osat = Oxygen saturation, TP = total phosphorus, SiO_2_ = Silica, Vel = Flow velocity, NLR = Number of limiting nutrient. For historical factors: Glac = Proportion of time under ice sheet the last 35 ka per site. Plot designed with R function *corrplot::corrplot* (Wei & Simko, 2021).

Many predictor variables were significantly correlated, and mostly positively (Figure 3). A few notable correlations will be commented on briefly. The positive correlation between Elevation, Longitude, and Latitude is a consequence of the long shape of Chile and the Andean mountains in the east of the sampling area being taller in the north (Figure 1). Further, all climatical variables were positively correlated with Latitude. Regarding the physicochemical variables that exhibited significant, while In situ temperature (T_insitu), pH, and Silicia (SiO_2_) were correlated with Latitude, Oxygen saturation (Osat), and Flow velocity (Vel) did not show any relationship with spatial variables. Also, Number of limiting resources (NLR) and Proportion of time under ice sheet (Glac) showed a negative latitudinal pattern.

### 3.3 Model results

For the Complete dataset, all assessed models showed at least one significant predictor, except for the Historical effect equation and Species energy via temperature (Table 2). Models with a better performance (lower AIC) included Species energy via cell density (deviance explained = 9.87%), Climate effect, and Total effect. The latter both included the same predictors (annual mean temperature = T_mean, temperature seasonality = T_sd, and maximum temperature during the warmest month = T_max), with a similar proportion of the deviance explained (~14%).

For the Chemical dataset, many hypotheses and equations indicated significant predictors. However, the Total effect equation performed best, explaining 41% of the deviance with the lowest AIC (= 1100.17) (Table 2). The second‐best result according to the deviance explained (30%) was for the Chemical effect equation, whereas the second‐best result according to the AIC was for the Niche dimensionality equation, with relatively high deviance explained (8%) for one variable. By contrast, historical factors showed no significant predictors, and Metabolic theory equations showed a significant relationship only for the Complete dataset and explained <2% of deviance, an effect size we consider biologically meaningless. The significant predictors in the Total effect equation were two climatical variables (T_max and T_mean) and four physicochemical variables (In situ temperature = T_insitu, Ph, Oxygen saturation = Osat, Silica = SiO_2_, and Flow velocity = Vel). Detailed results for each GAM are available in the Supplemental Material (Table S1).

**FIGURE 4:**
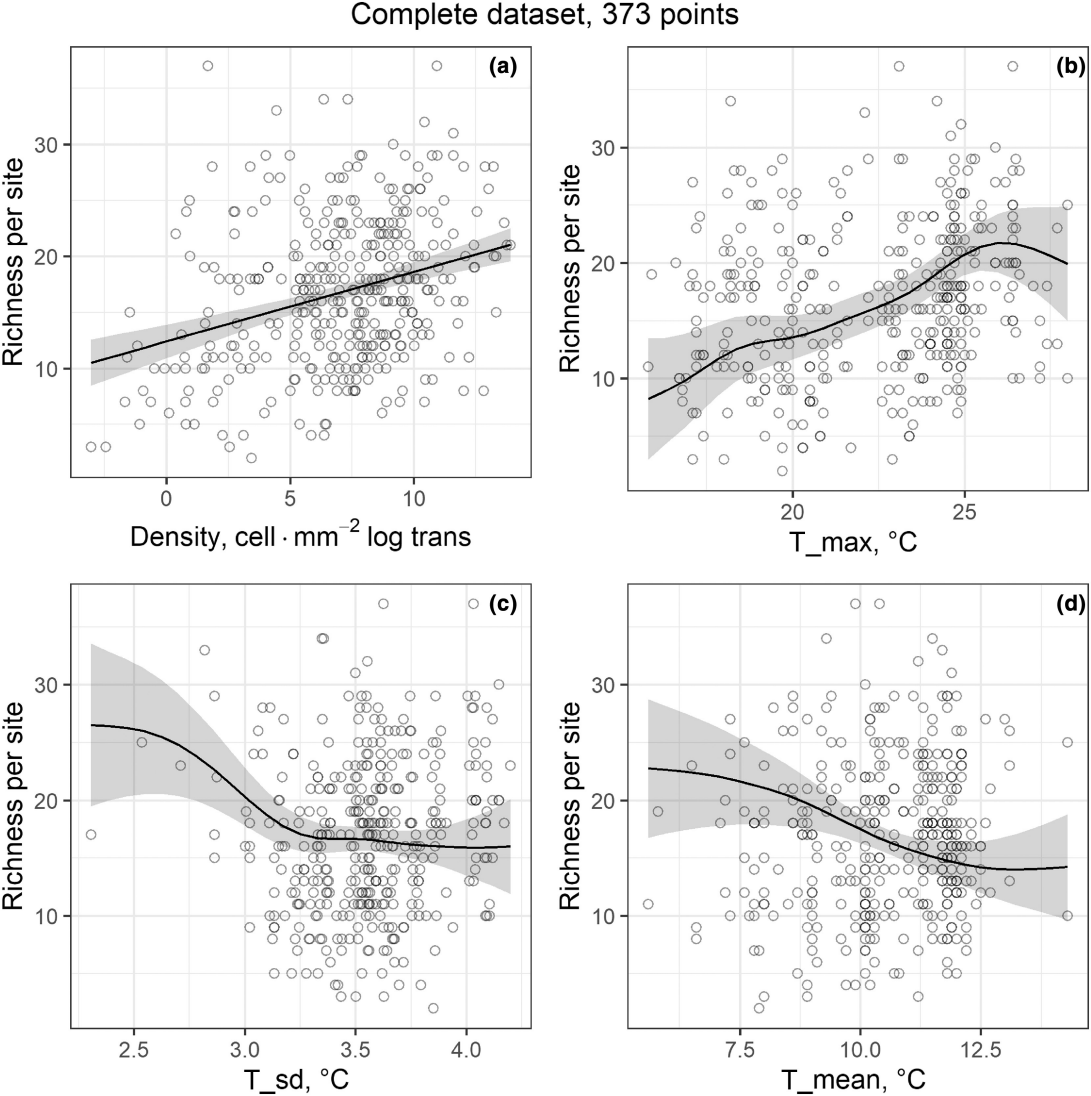
Single‐predictor relationships between species richness and the significant predictor variables from GAMs for the Complete dataset (373 points). The smoothed line represents the GAM prediction and the gray area its confident interval. Solid line = significant model. (a) Diatom richness in relation to cell density and smoothed line from Species‐energy theory model. (b) Species richness in relation to annual maximal temperature (T_max). (c) Species richness in relation to annual temperature seasonality (T_sd). (d) Species richness in relation to annual average temperature (T_mean). For b–d, smoothed splines are from the Climatical effect model. See text for more details.

**FIGURE 5:**
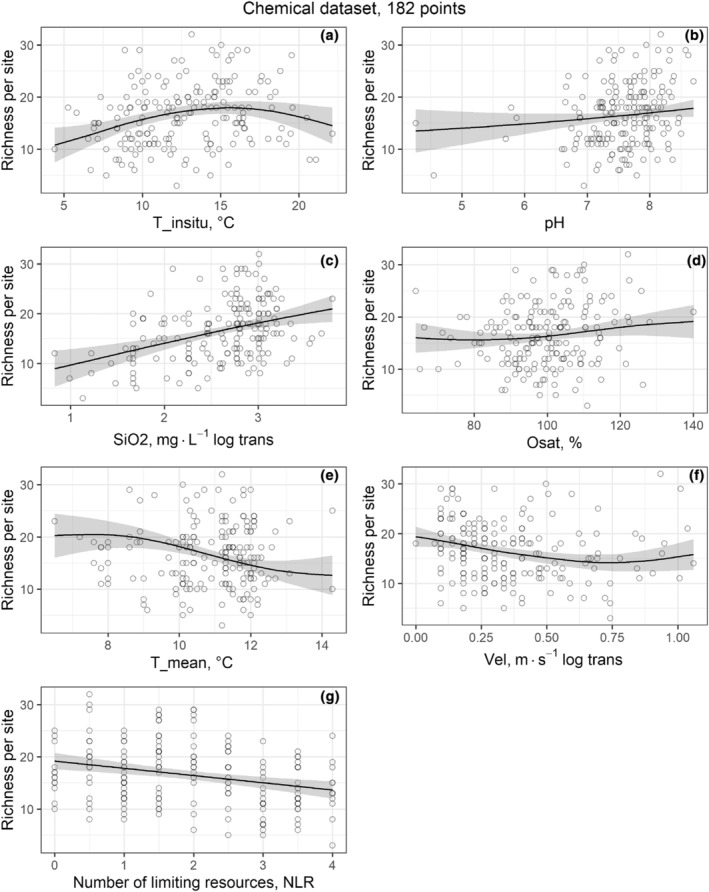
Single‐predictor relationships between species richness and the significant predictor variables from GAMs for the Chemical dataset (182 points). The smoothed line represents the GAM prediction and the gray area its confident interval. Solid line = significant model. Species richness is shown in relation to (a) in situ temperature (T_insitu), (b) pH, (c) silica (SiO_2_), (d) saturation oxygen (Osat), (e) annual mean temperature (T_mean), (f) flow velocity (Vel), and (g) number of limiting resources (NLR). a–f are smoothed splines from the total effect model and (g) is a linear model from the Niche dimensionality model.

Figures 4 and 5 show the single‐predictor relationships between diatom species richness and significant predictors, such as climatical, physicochemical, or biological (cell density) variables, illustrating the GAM results for their best models. For the Complete dataset, the plotted predictors were selected in the Species energy via cell density and Climatical effect models (Figure 4). Diatom richness significantly increased with cell density (Figure 4a), and also increased with T_max until about 27°C to then decrease at the upper end of the range (Figure 4b). Further, richness decreased with rising T_sd (Figure 4c) and T_mean (Figure 4d). For the Chemical dataset, the plotted predictors were selected in the Chemical effect, Total effect, and Niche dimensionality models (Figure 5). Diatom richness displayed a unimodal distribution with respect to T_insitu (Figure 5a), peaking at approximately 15°C. Also, richness increased with pH (Figure 5b), SiO_2_ (Figure 5c), and Osat (Figure 5d), and decreased with T_mean (Figure 5e), Vel (Figure 5f), and Number of limiting resources (Figure 5g).

Results for formal hypotheses are presented next, and the expected patterns for each model are listed in Table 1. The Species‐energy theory was validated for Cell density, with a positive relationship between Cell density and diatom richness (Figure 4a). However, the pattern for temperature did not fulfill the theoretical predictions, due to showing no significant relationship between diatom richness and T_mean in any dataset (Table 2). For the Energy variability hypothesis model, the deviance explained in both datasets was low, and the relationship between T_sd and diatom richness was U‐shaped for the Complete dataset (Figure S1a) and positive for Chemical dataset (Figure S1b), contrary to the negative pattern expected. The Climatical tolerance hypothesis also failed to fulfill the theoretical expectations: while the deviance explained was relatively high, the relationship detected between T_min and richness was U‐shaped and negative instead of positive (Figure S2a,c), and the relationship between T_max and richness were positive instead of negative (Figure S2b,d). Regarding the Metabolic theory model, we found no significant results for the Chemical dataset. For the Complete dataset, we found a significant positive relationship, but the deviance explained was too small to be ecologically relevant and the pattern observed was almost flat (Figure S3). For the Historical effect model, we found no significant results in any dataset (Table 2). Finally, for the Niche dimensionality model, the pattern detected fulfilled the theoretical expectations, with negative and significative relationship between richness and NLR (Figure 5g).

### 3.4 Metacommunity analysis

The results of the *metacom::Metacommunity* function suggested a coherent metacommunity, with significantly less embedded absences than null matrices (embAbs = 45,911, *z* = −3.01, *p* = .003). Turnover was significant and positive (turnover = 66,354,370, *z* = 7.31, *p* < .0001), reflecting the tendency for species to replace each other from site to site. Boundary clumping was also significant and positive (Morisita's index = 2.54, *p* < .0001), indicating that species shared similar distribution ranges. When all these parameters were applied into the framework by Presley et al. (2010), the framework indicated a Clementsian metacommunity structure in our case. Finally, when we correlated the order of each site in the reordered matrix versus their latitude, this correlation was significant (*t* = −3.08, df = 371, *p* = .002), but the same analysis versus their species richness showed no significant result (*t* = −0.30, df = 371, *p* = .77).

## 4 DISCUSSION

Diatom species richness showed a clear spatial structure in our study, increasing with decreasing latitude between 52° S and 35° S. Richness was also related to physicochemical variables (Silica and the number of limiting resources, NLR), and to climatical variables (Annual mean temperature and Maximum temperature during the warmest month). However, the relationship between species richness and climatical variables contrast strongly with the main theories proposed to explain biogeographical patterns, such as Energy variability hypothesis or Climatical tolerance hypothesis. For example, richness should increase with mean temperature, but it decreased in our study, and richness should decrease with maximum temperature but whereas it mostly increased in our study. On the other hand, our results are broadly consistent with alternative hypotheses put forward to explain geographical patterns specifically in stream diatoms based on physicochemical parameters (Passy, 2010; Passy et al., 2018; Soininen, 2007; Soininen et al., 2016).

### 4.1 Spatial patterns of diatoms in southwestern South America

If we compare the northward richness increase pattern with other geographic patterns for diatoms, our results do not match those in any other regional‐level study (Passy, 2010; Passy et al., 2018). However, they parallel the general increase of diatom species richness towards the Northern hemisphere found by Soininen et al. (2016). Regarding the diatom data reported by Soininen et al. (2016), the maximum species richness of other regions located in the Southern hemisphere is similar to our own findings (Chile = 37; New Zealand ~40; Reunion ~50). By contrast, these numbers are lower than the maximum richness of Northern hemisphere regions in Soininen et al. (~100 species) and in other related studies (~90 species) (Jamoneau et al., 2022; Passy, 2008, 2010; Passy et al., 2018).

Regarding the geographic patterns of other species groups, the pattern detected in our study is consistent with the latitudinal diversity gradient hypothesis (LDG) to some extent due to the richness increase towards the north. Also, the pattern matches the unimodal distribution recorded in Chile for many other organisms, with a species richness peak around 38° S (Cofré et al., 2007; Fernández et al., 2016; Segovia et al., 2013; Vila et al., 1999; Villagrán & Hinojosa, 1997). Regrettably, our dataset (collected between 35° S and 52° S latitude) does not allow testing how stream diatom richness pattern might change above 38° S south.

Because the northward richness increase pattern was not apparent when the data were split into latitudinal bands, we suggest that the diatom species pool is not larger in the north. Instead, we propose northern sites are able to support higher number of species because of local conditions. Northern sites are located on areas with higher human presence, which could determine sites with lower number of limiting resources and then higher species richness (Passy, 2008). Given that the relationship between historical variables (the proportion of time under ice sheet = Glac or presence/absence of ice sheet = Fglac) and the species richness was weak, and that a Nested metacommunity structure was discarded, the richness pattern recorded was probably not constrained by historical factors. Instead, it is likely to mostly depend on environmental conditions, which also explain the high variability in species richness detected in our study.

### 4.2 Diatom species richness patterns and formal hypotheses

In general, models related to climate theories revealed inconsistent results. For the Climatic tolerance hypothesis (Currie et al., 2004), our results were upside down for the annual maximal temperature, with a strong positive pattern instead of a negative one, and results were inconsistent for annual minimal temperature, with u‐shaped and positive patterns instead of negative ones. Regarding the Energy variability theory, we observed no consistent relationship between species richness and temperature seasonality, thus failing to support the consistently negative relationship predicted by this theory (Carrara & Vázquez, 2010). We also found no evidence to support the Metabolic theory of ecology (MTE). This result is similar to several other studies, both for stream diatoms (Passy et al., 2018) and for other taxonomic groups (Hawkins et al., 2007; Segura et al., 2015). We note that for many other taxonomic groups, climate theories were able to account for approximately 60% of the observed variance in species richness (Carrara & Vázquez, 2010; Rodríguez et al., 2005; Spasojevic et al., 2014). This marked difference from our dataset, where the proportion of variance explained was much lower (2%–11%) and the obtained patterns did not match predictions, highlights the fact that stream diatoms are indeed one exception to this rule.

For the Species‐energy theory tested via temperature, the relationship between Annual mean temperature (T_mean) and species richness was non‐significant, contrary to the positive pattern expected (Wright, 1983). Further, although the relationship between latitude and T_mean was positive, and the relationship between latitude and species richness was also positive, the relationship between T_mean and species richness was slightly negative for the Climatical effect model. When combined, these results reject the species‐energy theory tested via temperature for our study and suggest that the idea of latitude driving temperature to control species richness can also be rejected. However, the equation for the species‐energy theory tested via cell density was significantly positive for both datasets. These contradictory results for T_mean and cell density make it difficult to interpret our findings in terms of the species‐energy theory. Therefore, we suggest that the observed distribution patterns may be attributed to the role of the three‐dimensional benthic periphyton mat. This mat allows for a simultaneous increase in cell density and richness (Hillebrand, 2003; Passy, 2008), without water temperature playing an important role. Overall, the lack of results to support climate theories, and the importance of physicochemical variables in several equations, including the significance of the Niche dimensionality equation (Passy, 2008), allow us to suggest that diatom species richness is explained better through nutrient supply than theories related to climatical variables.

We found that the regression model accounting for the highest proportion of variance in diatom species richness included both climatical and chemical predictor variables. When combined, these results suggest that the principal drivers of diatom richness patterns were physicochemical variables, some of which exhibited a spatial pattern mediated by environmental variables, but some did not. Silica (SiO_2_) and pH increased with latitude and temperature in situ (T_insitu) was correlated with environmental temperature (T_mean), a variable that increases with latitude. By contrast, flow velocity (Vel) and Oxygen saturation (Osat) showed no clear spatial patterns. Consequently, the geographical patterns shown by the species richness of stream diatoms across southwestern South America can be described reasonably well by the spatial distribution of pH and Silica. At the same time, the high variability of this pattern may be explained by the lack of spatial constrains for other important physicochemical predictors such as Flow velocity and Oxygen saturation.

### 4.3 Global geographical patterns in stream diatoms

Our results are broadly consistent with the model proposed by Passy (2008) and Passy (2016) and the results of Passy (2010) and Passy et al. (2018), which all concluded that nutrient supply was the main driver of diatom richness. However, in these three studies (which used data from the USA and Finland) and two studies based on a worldwide database (Jamoneau et al., 2022; Soininen et al., 2016), the most important chemical variables (pH, electrical conductivity, iron, or phosphorus) differed from the ones in our study variables (In situ temperature, pH, Oxygen saturation, Silica, and Flow velocity). These differences highlight the relevance of natural and anthropogenic variability in key nutrients for stream diatoms at regional scales across the world, which prevents finding a single geographical pattern for these organisms. Consequently, to advance our understanding of the drivers of richness patterns in stream diatoms, perhaps we should use more frequently a single metric of nutrient supply, as suggested by Passy (2008), and then complement this nutrient metric by adding new metrics related to historical factors. These metrics could be the Proportion of time under ice sheet (as in our study) or dispersal processes of diatoms, as in Jamoneau et al. (2018) and Rusanov and Khromov (2016). The latter two studies included distance between sampling points to estimate colonization probability between sites.

Further, the concept of viewing nutrient supply as the main driver of diatom species richness needs to be complemented by the role of historical and dispersal processes in freshwater algae (Bennett et al., 2010; Soininen et al., 2016; Verleyen et al., 2009). In our case, a possible impact of the Last Maximum Glaciation limiting species richness was discarded. First, variables related to glaciation were never significant or selected by statistical models over physicochemical variables. Secondly, dispersal limitations determined by historical process should determine a Nested metacommunity (Srinivasan et al., 2014), but this did not occur in our data. Instead, our results showed a Clementsian metacommunity structure which changed significantly with latitude, but this change was unrelated to species richness. This result suggests clustered diatom communities likely shaped by environmental conditions, as discussed earlier.

## 5 CONCLUSION

We observed an increase of diatom species richness from south to north. This pattern differed from that detected in regional benthic diatom studies outside of South America but is consistent with a global study (Soininen et al., 2016). Physicochemical variables were the best predictors of diatom richness, with Silica and Flow Velocity being the most influential. Silica exhibited a clear spatial pattern that increased towards the north, while Flow velocity showed no obvious spatial pattern. This duality likely contributed to both the observed increase in species richness towards the north and the high variability in the diatom richness pattern. Niche dimensionality theory provided the most likely explanation for the richness pattern detected, according to which theory sites with higher nutrient levels can support higher species richness through enabling the development of complex algal biofilms (Passy, 2008). Our findings lend further weight to previous related research from other regions of the world, which demonstrates that ecological theories developed based on terrestrial datasets cannot explain the biogeographical patterns of freshwater diatom communities. Therefore, a new framework is needed to explain biogeographical patterns in this important group of freshwater organisms.

## AUTHOR CONTRIBUTIONS

**Daniel Zamorano:** Conceptualization (equal); data curation (equal); formal analysis (lead); investigation (equal); methodology (equal); supervision (equal); writing – original draft (equal); writing – review and editing (equal). **Fabio A. Labra:** Conceptualization (equal); investigation (equal); methodology (equal); supervision (lead); writing – original draft (equal); writing – review and editing (equal). **Christoph D. Matthaei:** Investigation (equal); methodology (equal); writing – original draft (equal); writing – review and editing (lead). **Úrsula Romero:** Conceptualization (equal); data curation (lead); investigation (equal); methodology (equal); project administration (lead); writing – review and editing (supporting).

## CONFLICT OF INTEREST STATEMENT

The authors declare no conflicting interests.

## Supporting information


Appendix S1.

## Data Availability

The raw data of this study, including environmental variables and taxonomic sheet, plus the R code with all models and graphs, were included as online supplement at the following link: https://figshare.com/s/f550bf7624f90a6fe81a.
