# Supplementary material for: Biogeographical patterns of species richness in stream diatoms from southwestern South America
Source: Ecol Evol. 2024 Mar 20;14(3):e11156. doi: 10.1002/ece3.11156 (PMC10954374; doi:10.1002/ece3.11156)
Supplement: Supplementary file 1 — Appendix S1. [file ECE3-14-e11156-s001.docx]

**Table S1****.** Detailed results of GAM models for both datasets per predictor variable. For all predictors without an “s” in their name, the model was linear and degree of freedom (df) = 1. All predictor variables with an “s” were smooth-spline transformed, and “df” are estimated to penalize this transformation.

| Group | Model | var | df | F | pval | sig |
| --- | --- | --- | --- | --- | --- | --- |
| All | Latitudinal effect | s(lat) | 3.57E+00 | 4.08E+00 | 2.24E-08 | *** |
| All | Space effect | s(elevation) | 8.62E-01 | 6.92E-01 | 6.03E-03 | ** |
| All | Space effect | s(lat) | 1.96E+00 | 2.12E+00 | 1.61E-05 | *** |
| All | Space effect | s(lon) | 5.41E-04 | 3.14E-05 | 4.90E-01 |  |
| All | Species-energy theory via temperature | s(t_mean) | 7.07E-01 | 2.67E-01 | 6.61E-02 | . |
| All | Species-energy theory via cell density | s(abund) | 9.78E-01 | 4.41E+00 | 5.08E-10 | *** |
| All | Energy variability hypothesis | s(t_sd) | 2.60E+00 | 1.90E+00 | 1.59E-04 | *** |
| All | Climatical tolerance hypothesis | s(t_min) | 2.75E+00 | 1.02E+00 | 1.10E-02 | * |
| All | Climatical tolerance hypothesis | s(t_max) | 4.36E+00 | 4.22E+00 | 2.88E-08 | *** |
| All | Climatical effect | s(t_min) | 8.90E-03 | 8.68E-04 | 1.58E-01 |  |
| All | Climatical effect | s(t_max) | 3.80E+00 | 2.84E+00 | 5.50E-07 | *** |
| All | Climatical effect | s(t_sd) | 3.24E+00 | 1.60E+00 | 6.74E-04 | *** |
| All | Climatical effect | s(t_mean) | 2.03E+00 | 8.98E-01 | 3.54E-03 | ** |
| All | Historical effect | glac2 | 1 | 2.73E+00 | 9.94E-02 | . |
| All | Historical effect | fglac | 1 | 2.81E-02 | 8.67E-01 |  |
| All | Total effect | glac2 | 1 | 2.31E+00 | 1.29E-01 |  |
| All | Total effect | fglac | 1 | 1.31E-01 | 7.18E-01 |  |
| All | Total effect | s(t_min) | 2.97E-03 | 2.16E-04 | 3.11E-01 |  |
| All | Total effect | s(t_max) | 3.90E+00 | 1.79E+00 | 2.08E-04 | *** |
| All | Total effect | s(t_sd) | 3.08E+00 | 8.17E-01 | 2.73E-02 | * |
| All | Total effect | s(t_mean) | 1.36E+00 | 3.50E-01 | 5.03E-02 | . |
| All | Niche dimensionality | NLR | 1 | 1.54E+01 | 1.27E-04 | *** |
| All | Metabolic theory | 1/k*t_mean | 1 | 5.86E+00 | 1.59E-02 | * |
| Chem | Latitudinal effect | s(lat) | 2.14E+00 | 1.72E+00 | 2.54E-04 | *** |
| Chem | Space effect | s(elevation) | 3.52E-01 | 6.04E-02 | 1.66E-01 |  |
| Chem | Space effect | s(lat) | 1.28E+00 | 4.73E-01 | 3.46E-02 | * |
| Chem | Space effect | s(lon) | 8.30E-01 | 5.42E-01 | 4.30E-03 | ** |
| Chem | Species-energy theory via temperature | s(t_mean) | 7.01E-04 | 2.94E-06 | 1.00E+00 |  |
| Chem | Species-energy theory via cell density | s(abund) | 9.64E-01 | 2.94E+00 | 4.20E-07 | *** |
| Chem | Energy variability hypothesis | s(t_sd) | 9.17E-01 | 1.22E+00 | 6.54E-04 | *** |
| Chem | Climatical tolerance hypothesis | s(t_min) | 1.76E+00 | 1.14E+00 | 1.33E-03 | ** |
| Chem | Climatical tolerance hypothesis | s(t_max) | 2.10E+00 | 2.49E+00 | 4.04E-06 | *** |
| Chem | Climatical effect | s(t_min) | 1.24E+00 | 2.75E-01 | 6.31E-02 | . |
| Chem | Climatical effect | s(t_max) | 2.30E+00 | 2.06E+00 | 6.82E-06 | *** |
| Chem | Climatical effect | s(t_sd) | 3.94E-04 | 1.15E-05 | 7.37E-01 |  |
| Chem | Climatical effect | s(t_mean) | 5.84E-01 | 1.56E-01 | 2.19E-02 | * |
| Chem | Chemical effect | s(temp) | 2.02E+00 | 8.75E-01 | 1.00E-02 | * |
| Chem | Chemical effect | s(ph) | 9.87E-01 | 5.16E-01 | 1.97E-02 | * |
| Chem | Chemical effect | s(CE) | 4.41E-03 | 4.59E-04 | 3.34E-01 |  |
| Chem | Chemical effect | s(Osat) | 1.34E+00 | 5.04E-01 | 2.76E-02 | * |
| Chem | Chemical effect | s(Ca) | 5.42E-04 | 3.69E-05 | 4.76E-01 |  |
| Chem | Chemical effect | s(P_tot) | 3.29E-04 | 8.63E-06 | 7.62E-01 |  |
| Chem | Chemical effect | s(SiO2) | 1.39E+00 | 1.88E+00 | 2.42E-05 | *** |
| Chem | Chemical effect | s(vel_fon) | 1.92E+00 | 1.31E+00 | 1.00E-03 | ** |
| Chem | Total effect | s(temp) | 2.32E+00 | 1.80E+00 | 9.97E-05 | *** |
| Chem | Total effect | s(ph) | 8.71E-01 | 3.64E-01 | 3.89E-02 | * |
| Chem | Total effect | s(CE) | 1.39E-04 | 8.44E-06 | 4.77E-01 |  |
| Chem | Total effect | s(t_min) | 1.08E-04 | 3.23E-06 | 6.53E-01 |  |
| Chem | Total effect | s(t_max) | 1.75E+00 | 4.54E-01 | 5.06E-02 | . |
| Chem | Total effect | s(t_sd) | 8.01E-05 | 1.37E-06 | 8.67E-01 |  |
| Chem | Total effect | s(t_mean) | 1.92E+00 | 1.55E+00 | 7.46E-05 | *** |
| Chem | Total effect | s(Osat) | 1.31E+00 | 4.46E-01 | 3.76E-02 | * |
| Chem | Total effect | s(Ca) | 5.44E-01 | 9.19E-02 | 1.94E-01 |  |
| Chem | Total effect | s(P_tot) | 4.19E-05 | 6.03E-07 | 9.35E-01 |  |
| Chem | Total effect | s(SiO2) | 1.24E+00 | 1.64E+00 | 4.16E-05 | *** |
| Chem | Total effect | s(vel_fon) | 1.92E+00 | 1.57E+00 | 2.33E-04 | *** |
| Chem | Historical effect | glac2 | 1 | 3.56E+00 | 6.09E-02 | . |
| Chem | Historical effect | fglac | 1 | 1.46E+00 | 2.29E-01 |  |
| Chem | Niche dimensionality | NLR | 1 | 1.54E+01 | 1.27E-04 | *** |
| Chem | Metabolic theory | 1/k*t_mean | 1 | 8.45E-02 | 7.72E-01 |  |


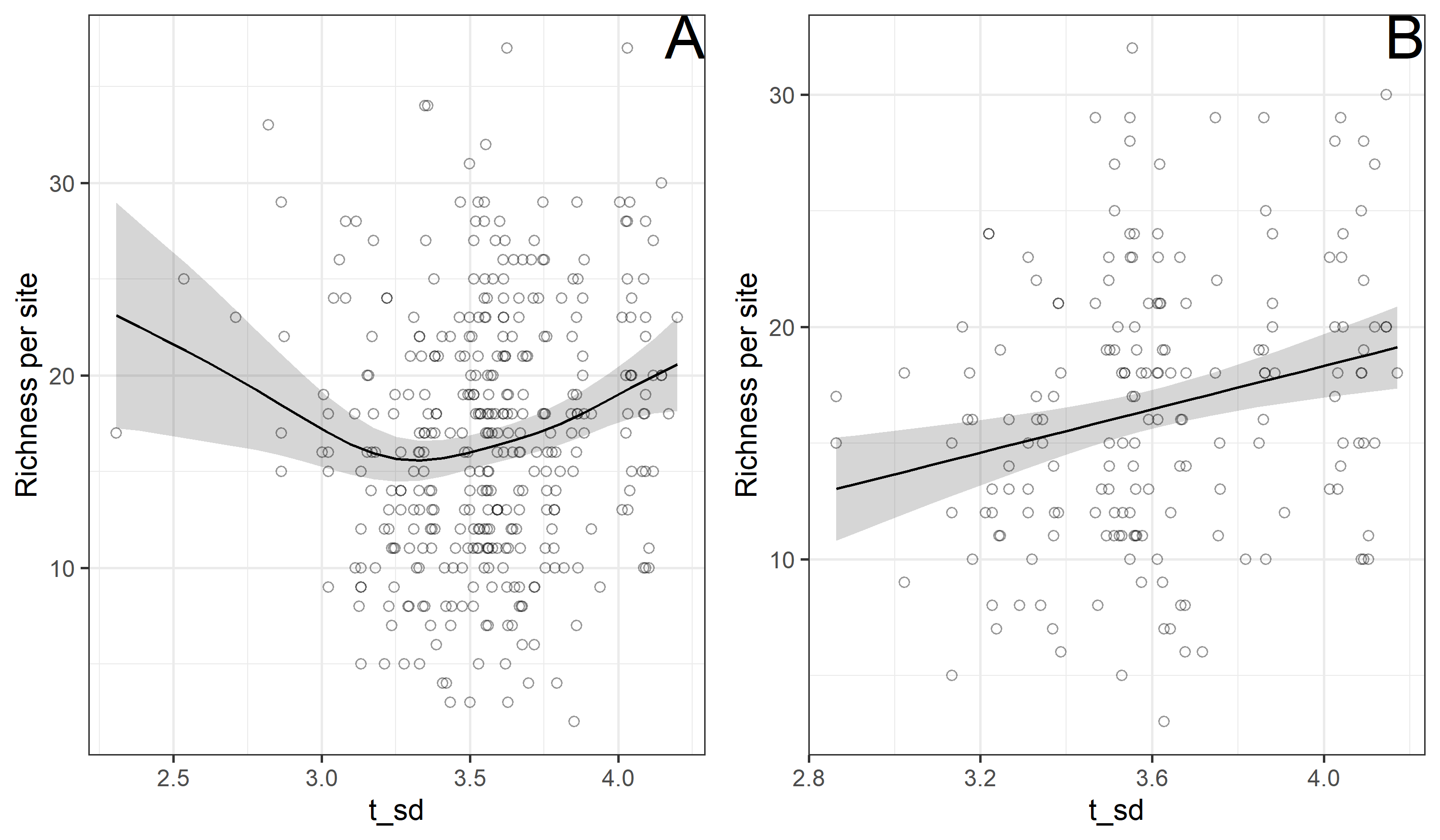


**Figure S1.** Single-predictor relationships between species richness and temperature seasonality (t_sd) for the ‘Energy variability hypothesis’ model. A, Model for the Complete dataset. B, Model for the Chemical dataset. The smoothed line represents the GAM prediction and the gray area its confidence interval.


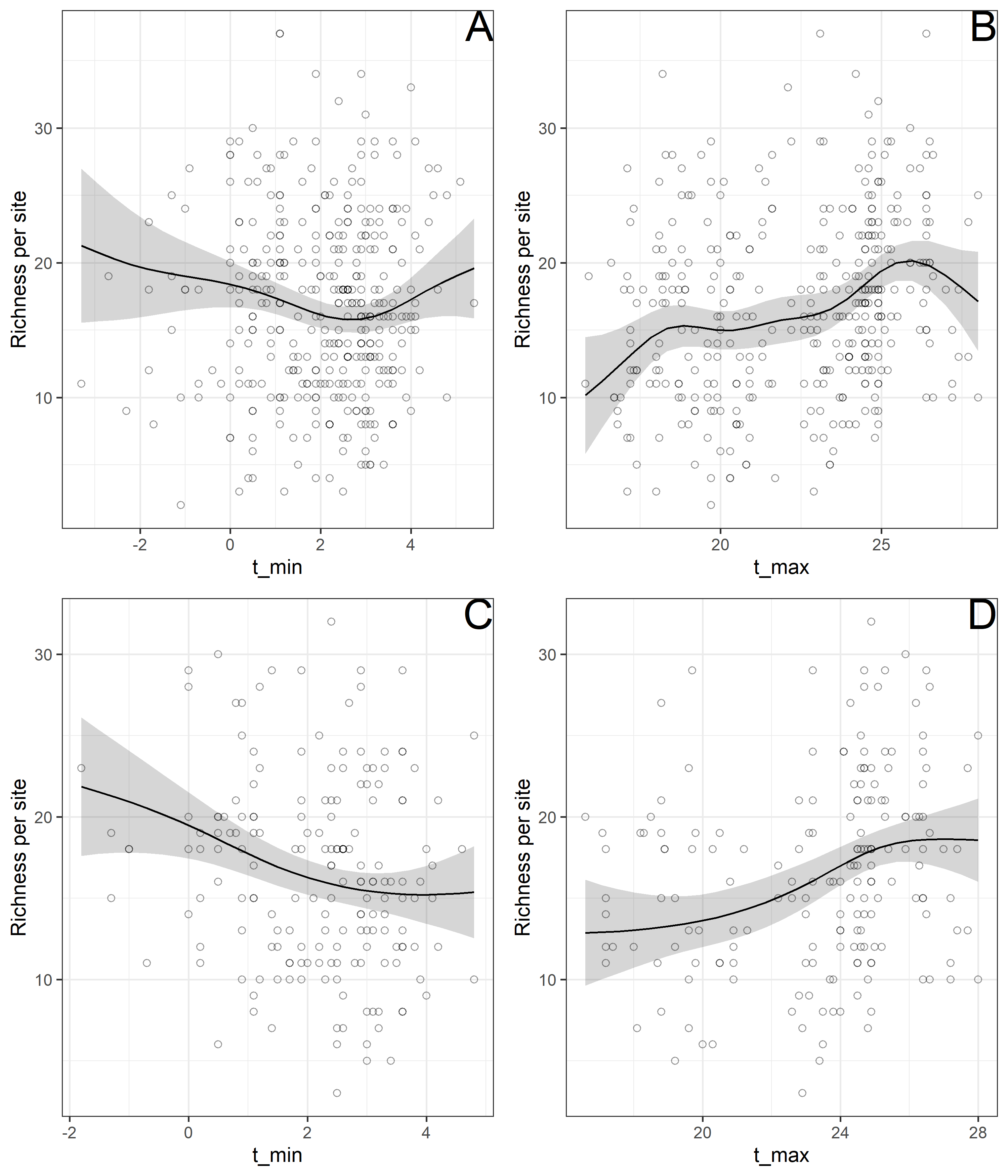


**Figure S2.** Single-predictor relationships between species richness and predictors for the ‘Climatical tolerance hypothesis’ model. A, Model for the Complete dataset for annual minimal temperature (T_min). B, Model for the Complete dataset for annual maximal temperature (T_max). C, Chemical dataset model for T_min. D, Chemical dataset model for T_max. The smoothed line represents the GAM prediction and the gray area its confidence interval.


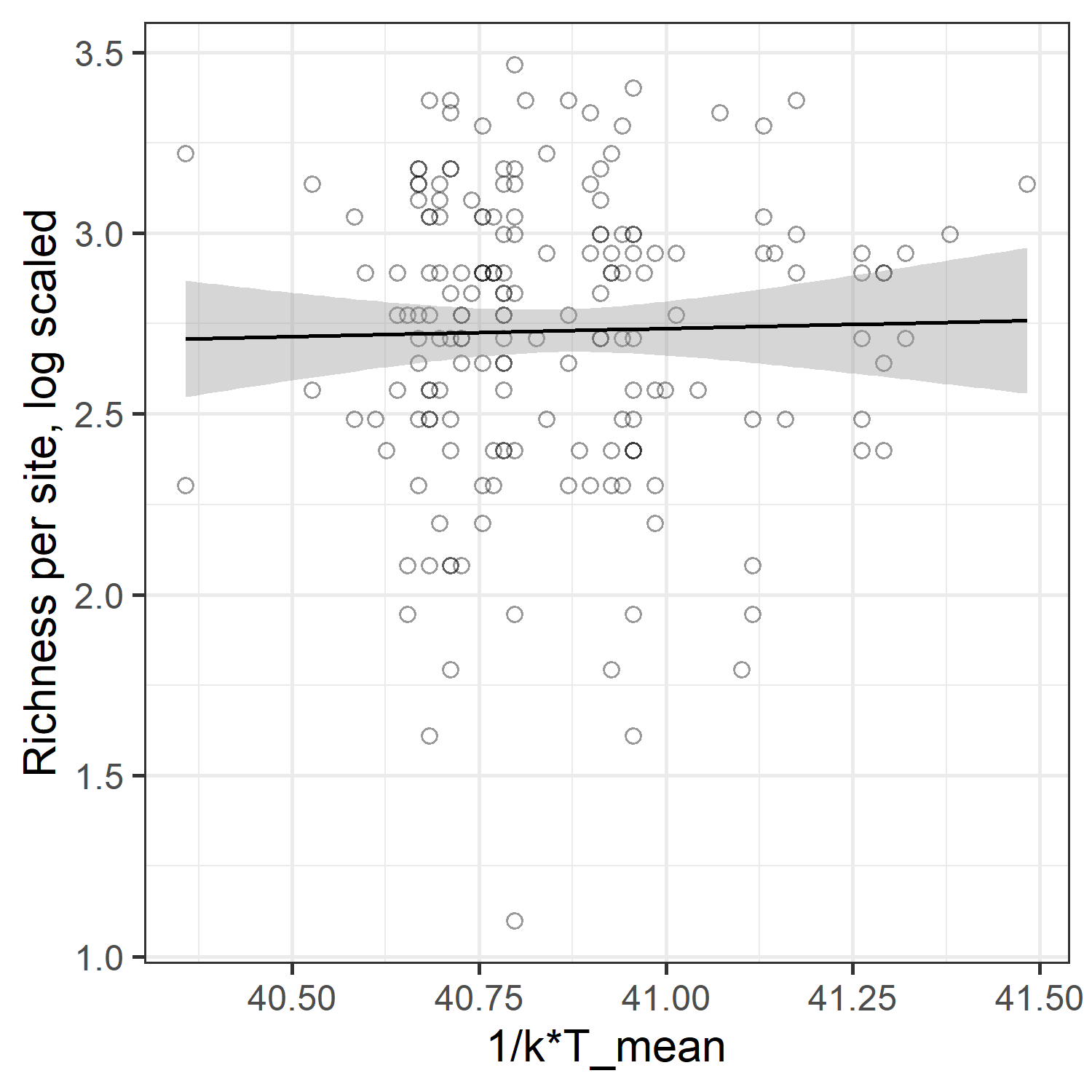


**Figure S3.** Single-predictor relationships between species richness and energy for the ‘Metabolic theory’ model. Energy was estimated by a 1/k*T_mean transformation, where T_mean is annual average temperature (see Methods for more details). The smoothed line represents the GAM prediction and the gray area its confidence interval.
